# Supplementary material for: Species abundance correlations carry limited information about microbial network interactions
Source: PLoS Comput Biol. 2022 Sep 9;18(9):e1010491. doi: 10.1371/journal.pcbi.1010491 (PMC9518925; doi:10.1371/journal.pcbi.1010491)
Supplement: S1 Text — Table A. Conditions for stable co-existence in the two-species Lotka-Volterra model. Fig A. Zero-growth isoclines (“null-clines”) in the two-species Lotka-Volterra model. (PDF) [file pcbi.1010491.s001.pdf]

S1 Text. Co-existence in a two-species Lotka-Volterra model with self-limitation

The conditions for co-existence in the two-species Lotka-Volterra model with self-limitation can be derived by setting both growth equations to zero and investigating what parameter combinations yield $\bar{N}_1 > 0$ as well as $\bar{N}_2 > 0$. Here, $\bar{N}_1$ denotes the equilibrium abundance of species 1 and $\bar{N}_2$ denotes the equilibrium abundance of species 2. Writing these conditions in terms of N_1 as functions of N_2 gives the following:

$$\frac{dN_1}{dt} = 0 \wedge N_1 > \ 0 \ \rightarrow N_1 = f_1(N_2) = K_1 + \alpha_{12}K_1N_2 \quad \text{Eq. 1}$$

$$\frac{dN_2}{dt} = 0 \wedge N_2 > \ 0 \ \rightarrow N_1 = f_2(N_2) = \ -\frac{1}{\alpha_{21}} + \frac{1}{(\alpha_{21}K_2)}N_2 \quad \text{Eq. 2}$$

The joint equilibrium abundance of both species ($\bar{N}_1$, $\bar{N}_2$) is determined by $f_1(N_2)$ = $f_2(N_2)$. Equation (Eq. 1) shows that species 1 grows to its carrying capacity K_1 in the absence of interspecific interactions, i.e. if $\alpha_{12} = 0$. Likewise, $\alpha_{12} > 0$ allows species 1 to grow to higher abundance in the presence of species 2 than determined by its own carrying capacity, whereas $\alpha_{12} < 0$ leads to a reduced abundance of species 1 in the presence of species 2. Similar relations hold for the abundance of species 2 in presence of species 1, depending on α_{21}. From Equation (Eq. 2), it can also be derived that $\bar{N}_1 > 0$ is only compatible with $\bar{N}_2$ being above its carrying capacity K_2 if at the same time $\alpha_{21} > 0$, whereas $\bar{N}_2$ being below K_2 requires $\alpha_{21} < 0$.

Joint inspection of equations (Eq. 1) and (Eq. 2) also establishes the following, more subtle, conditions for co-existence:

1. If $\alpha_{12} > 0$ and $\alpha_{21} > 0$, i.e., in case of mutualism, f_2 has a negative intercept in the Cartesian (N_2, N_1) coordinate system (Figure A (panel A) in S1 Text). As both functions have a positive slope in this situation, and f_1 always has a positive intercept, f_2 must have a stronger slope than f_1 for both to intersect in the positive

quadrant. This boils down to $\frac{1}{\alpha_{21}K_2} > \alpha_{12}K_1$, or equivalently $\alpha_{21}\alpha_{12} < \alpha_{11}\alpha_{22}$, as $K_i = -\frac{1}{\alpha_{ii}}$ by definition. This means that the product of interspecific mutualism needs to be smaller than the product of intraspecific competition for both species to co-exist, otherwise there is no control of population growth.

2. If $\alpha_{12} < 0$ and $\alpha_{21} < 0$, i.e., in case of competition, both functions have positive intercept and negative slope (Figure A (panel B) in S1 Text). Intersection in the positive quadrant requires the function with the larger intercept to intersect the abscissa, i.e., the N_2 axis where N_1 = 0, at a smaller value than the function with the smaller intercept. Thus, this requires $|\alpha_{21}| > \frac{1}{K_1}$ and $|\alpha_{12}| > \frac{1}{K_2}$, with f_1 having the larger intercept, or alternatively, $|\alpha_{21}| < \frac{1}{K_1}$ and $|\alpha_{12}| < \frac{1}{K_2}$, with f_2 having the larger intercept. In the first instance, interspecific competition is stronger than intraspecific competition, whereas in the second instance, interspecific competition is less strong than intraspecific competition. It turns out that only the last of these conditions yields a stable equilibrium, meaning that the abundances of both species return to equilibrium after small displacements.
3. If $\alpha_{12} < 0$ and $\alpha_{21} > 0$, i.e., in case of exploitation of species 1 by species 2, f_1 has a positive intercept and negative slope, whereas f_2 still has a negative intercept and positive slope (Figure A (panel C) in S1 Text). Intersection in the positive quadrant requires f_1 to intersect the abscissa at a larger value than K_2, the point where f_2 intersects the abscissa. The condition for co-existence thus becomes $|\alpha_{12}| < \frac{1}{K_2}$, or equivalently $\alpha_{12} < \alpha_{22}$, meaning that the parasite should exert stronger inhibitory effect on growth of oneself than on that of the exploited species.
4. Conversely, in case of exploitation of species 2 by species 1, i.e., if $\alpha_{12} > 0$ and $\alpha_{21} < 0$, both f_1 and f_2 have a positive intercept, but f_1 now has a positive slope whereas f_2 has a negative slope (Figure A (panel D) in S1 Text). Intersection in the positive

quadrant then requires f_1 to have a smaller intercept than f_2. The condition for co-existence thus becomes $|\alpha_{21}| < \frac{1}{K_1}$, or equivalently $\alpha_{21} < \alpha_{11}$, again meaning that the parasite should exert stronger inhibitory effect on growth of oneself than on that of the exploited species.

The additional requirement for stable co-existence is that the two-species system should be locally stable around the equilibria ($\overline{N}_1$, $\overline{N}_2$), which can be formalized in terms of the Jacobian matrix of the Lotka-Volterra model evaluated at ($\overline{N}_1$, $\overline{N}_2$). This amounts to determining trace and determinant of the matrix of the partial derivatives of the growth equations regarding either species, i.e.,

$$\begin{bmatrix} r_1 - 2r_1\overline{N}_1 / K_1 + r_1\alpha_{12}\overline{N}_2 & r_1\alpha_{12}\overline{N}_1 \\ r_2\alpha_{21}\overline{N}_2 & r_2 - 2r_2\overline{N}_2 / K_2 + r_2\alpha_{21}\overline{N}_1 \end{bmatrix}$$

It can be verified that the conditions for co-existence stated under mutualism and exploitative interactions yield equilibria that are locally stable, just as the last of the conditions under competition. We will not derive these conditions here, as these are covered by textbooks on theoretical ecology [1]. In summary, the two-species Lotka-Volterra model with self-limitation has the following possibilities for stable co-existence (Table A in S1 Text).

The condition for stable co-existence of competitors requires both species to have less effect on the growth of other species than on oneself. In case of an unstable equilibrium, either species will eventually outcompete the other; the species with initial advantage will drive the other species to extinction, a condition referred to as competitive exclusion [2, 3]. This will occur, for instance, when each species produces a substance which is toxic to the other species but relatively harmless to itself.

References

1. Yodzis P (eds). Introduction to theoretical ecology. Harper & Row Publishers, New York, 1989.

2. Gause GF. Experimental studies on the struggle for existence: 1. Mixed population of two species of yeast. Journal of Experimental Biology 1932; 9: 389–402.
3. Hardin G. The competitive exclusion principle. Science 1960; 131: 1292–1297

Table A. Conditions for stable co-existence in the two-species Lotka-Volterra model.

Type of interaction	Condition	Outcome
Mutualism<br>$\alpha_{12} > 0 \wedge \alpha_{21} > 0$	$\alpha_{12}\alpha_{21} < \frac{1}{K_1K_2}$	$\bar{N}_1 > K_1 \wedge \bar{N}_2 > K_2$
Competition<br>$\alpha_{12} < 0 \wedge \alpha_{21} < 0$	$\lvert\alpha_{12}\rvert < \frac{1}{K_2} \wedge \lvert\alpha_{21}\rvert < \frac{1}{K_1}$	$\bar{N}_1 < K_1 \wedge \bar{N}_2 < K_2$
Exploitative interaction type 1*<br>$\alpha_{12} < 0 \wedge \alpha_{21} > 0$	$\lvert\alpha_{12}\rvert < \frac{1}{K_2}$	$\bar{N}_1 < K_1 \wedge \bar{N}_2 > K_2$
Exploitative interaction type 2**<br>$\alpha_{12} > 0 \wedge \alpha_{21} < 0$	$\lvert\alpha_{21}\rvert < \frac{1}{K_1}$	$\bar{N}_1 > K_1 \wedge \bar{N}_2 < K_2$

** Exploitative interaction type 1: species 1 is being exploited by species 2*

*** Exploitative interaction type 2: species 2 is being exploited by species 1*

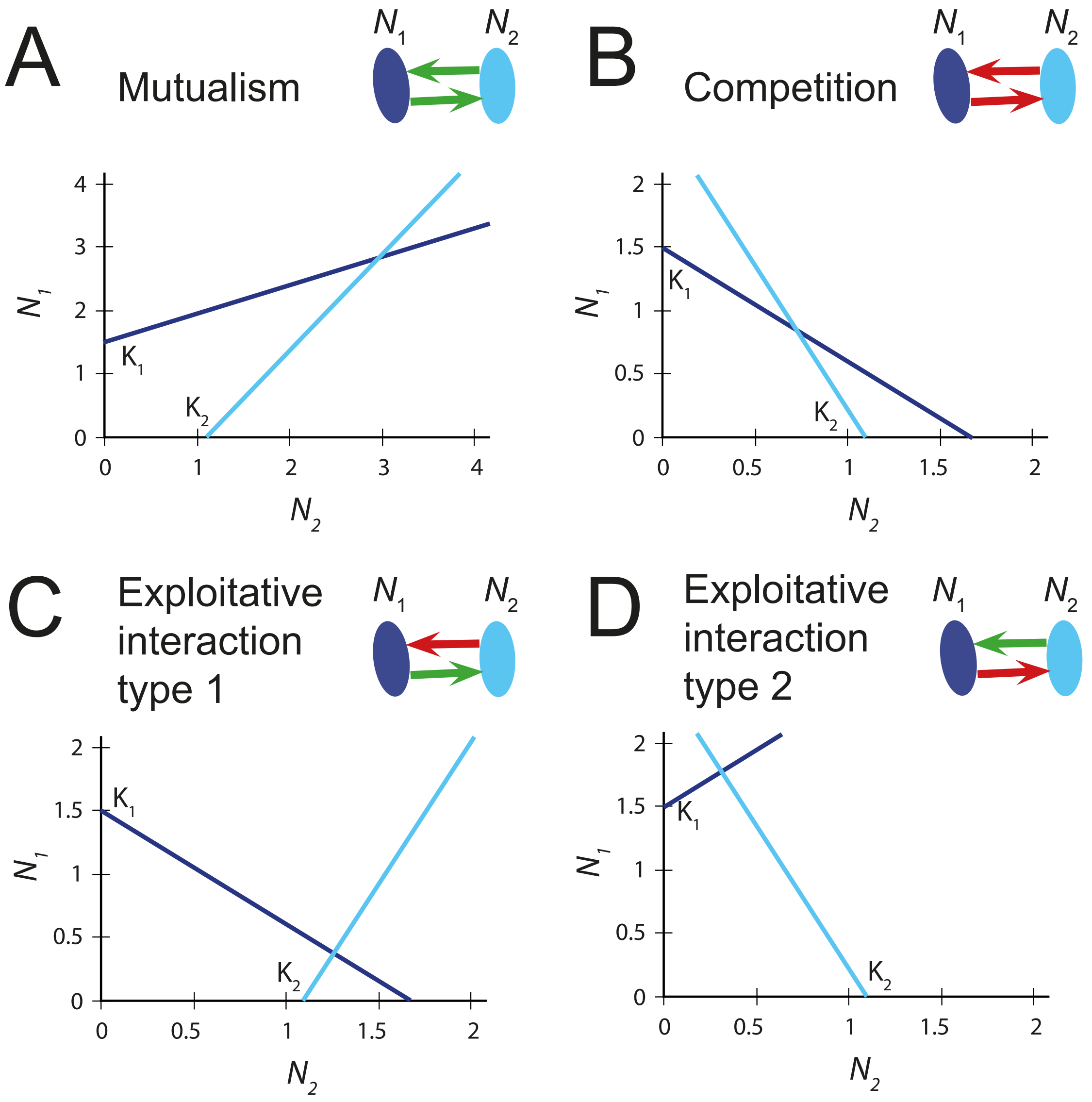

Figure A. Zero-growth isoclines ("null-clines") in the two-species Lotka-Volterra model. Visualization of the effect of species 1 and 2 abundance on each other in the Cartesian (N_2, N_1) coordinate system. Here, f_1 denotes the isocline of zero growth of species 1, i.e., $f_1(N_1)$ (in dark blue), and f_2 denotes the isocline of zero growth of species 2, i.e., $f_2(N_1)$ (in light blue). Their point of intersection represents the joint equilibrium abundance of both species, i.e. $(\bar{N}_2, \bar{N}_1)$. Throughout K_1= 1.5 and K_2= 1.1. Parameters for the various scenarios: α_{12} = 0.3 and α_{21} = 0.6 under mutualism; α_{12} = –0.6 and α_{21} = –0.4 under competition; α_{12} = –0.6 and α_{21} = 0.4 under exploitative interaction type 1; and α_{12} = 0.6 and α_{21} = –0.4 under exploitative interaction type 2.
