# Supplementary material for: Species abundance correlations carry limited information about microbial network interactions
Source: PLoS Comput Biol. 2022 Sep 9;18(9):e1010491. doi: 10.1371/journal.pcbi.1010491 (PMC9518925; doi:10.1371/journal.pcbi.1010491)
Supplement: S1 Fig — (PDF) [file pcbi.1010491.s002.pdf]

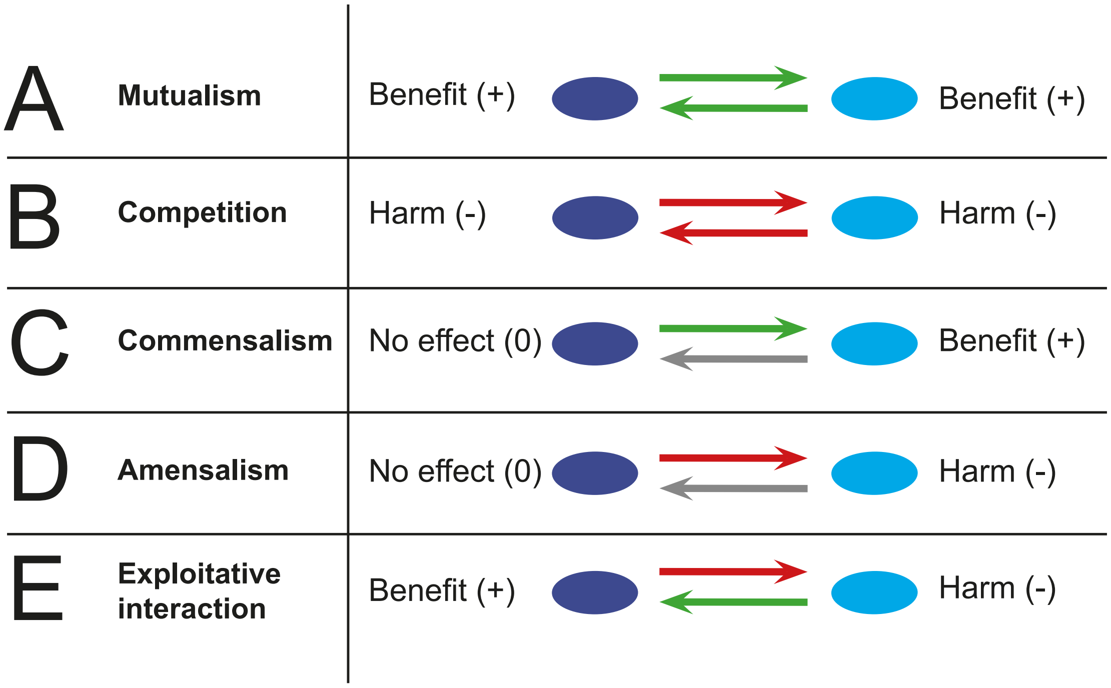

S1 Fig. Cartoon illustrating the different interaction mechanisms. (A) In mutualistic interactions, both species experience a positive effect. An example is when a species feeds on the metabolites excreted by the other species. (B) In competitive interactions both species experience a negative effect. An example is when both species produce toxic compounds that are harmful to the other species as well as to themselves. (C) Commensalism is a one-sided positive interaction. This type of interaction occurs when one species is beneficial to another species, without benefit or harm to itself. (D) Amensalism is a one-sided negative interaction. Amensalism occurs when a species causes harm to another species, without benefit or harm to itself. (E) Exploitative interactions occur when one species benefits from another species at the expense of the other. Red arrows represent negative interactions, green arrows represent positive interactions and grey arrows indicate no interactions.
