# Supplementary material for: Species abundance correlations carry limited information about microbial network interactions
Source: PLoS Comput Biol. 2022 Sep 9;18(9):e1010491. doi: 10.1371/journal.pcbi.1010491 (PMC9518925; doi:10.1371/journal.pcbi.1010491)
Supplement: S2 Fig — (PDF) [file pcbi.1010491.s003.pdf]

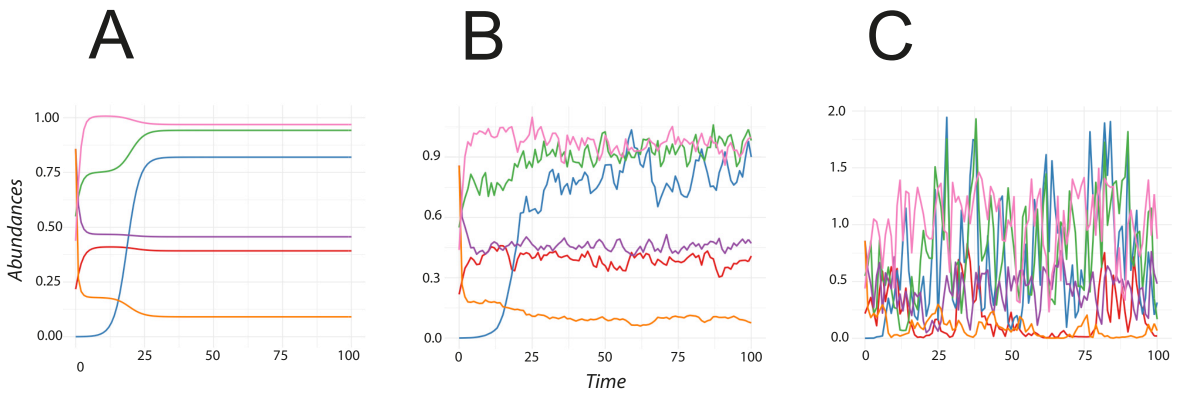

S2 Fig. The effect of process noise (*W*) on the within host population dynamics. Process noise was added by means of the "events" function from the deSolve package. The time-varying noise was drawn from a log-normal distribution to prevent the abundances from dropping below zero, i.e. $\Delta W_i = \ln(N_{i,m(\Delta t)}) - \ln(N_{i,m(t)}) \sim N(\ln(N_{i,t}), \sigma_W)$ at every timestep, $\Delta t = 1$. (A) Simulated timeseries without process noise, (B) with low process noise ($\sigma_W \sim N(0, 0.1)$) and (C) high process noise ($\sigma_W \sim N(0, 1)$).
