# Supplementary material for: Species abundance correlations carry limited information about microbial network interactions
Source: PLoS Comput Biol. 2022 Sep 9;18(9):e1010491. doi: 10.1371/journal.pcbi.1010491 (PMC9518925; doi:10.1371/journal.pcbi.1010491)
Supplement: S3 Fig — (PDF) [file pcbi.1010491.s004.pdf]

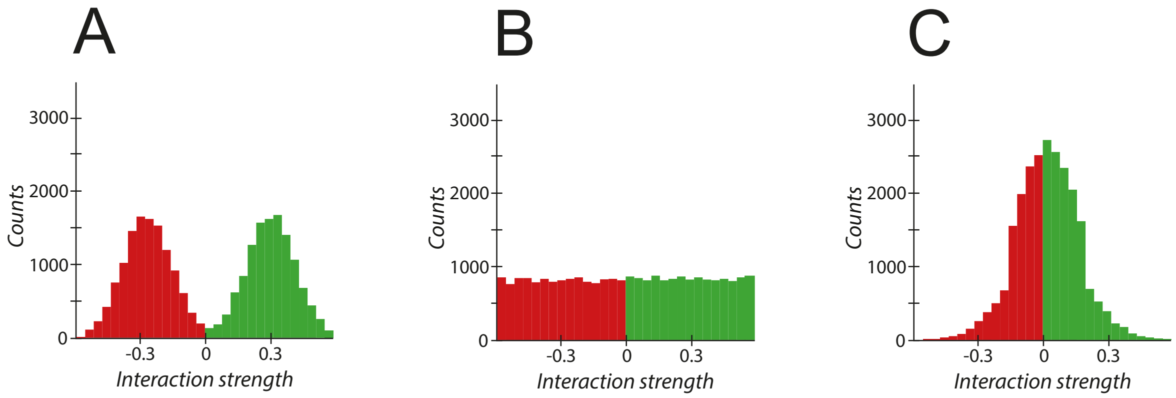

S3 Fig. Distributions of interaction strengths in three different scenarios. (A) The interaction strengths in the base case follow a Gaussian mixture distribution. Half of the interactions were drawn from a negative normal distribution: α_{ij} ~ N(–0.25, 0.1); and the other half of the interactions were drawn from a positive normal distribution: α_{ij} ~ N(0.25, 0.1). (B) The interactions strength in fig. 4D-1 follow a uniform distribution (α_{ij} ~ U(-0.5, 0.5)). (C) The interactions strength in fig. 4D-2 follow a unimodal distribution (α_{ij} ~ N(0, 0.15)). All interactions were restricted to lie between –0.5 and 0.5, i.e., the normal distributions were truncated at –0.5 and 0.5.
