# Supplementary material for: Species abundance correlations carry limited information about microbial network interactions
Source: PLoS Comput Biol. 2022 Sep 9;18(9):e1010491. doi: 10.1371/journal.pcbi.1010491 (PMC9518925; doi:10.1371/journal.pcbi.1010491)
Supplement: S4 Fig — (PDF) [file pcbi.1010491.s005.pdf]

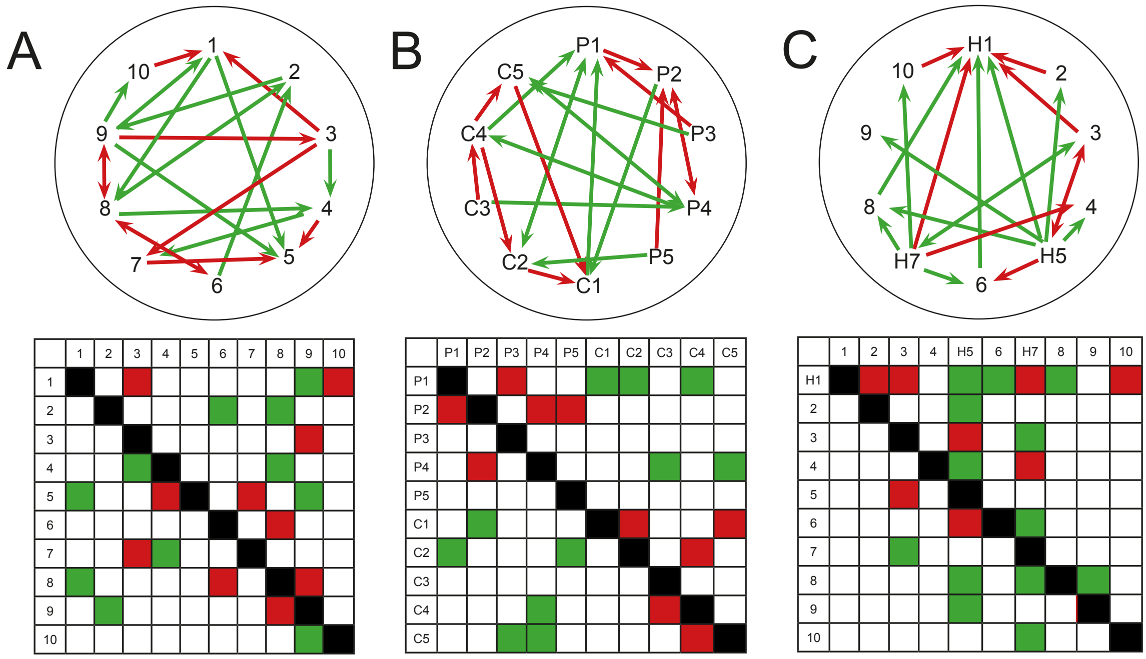

S4 Fig. Network structures used in the different case studies. (A) An example of a random network and its corresponding interaction matrix. (B) An example of a structured network with interaction modules and its corresponding interaction matrix. The modular networks are based on a cross-feeding structure between producers and consumers (with equal numbers of producers and consumers). Between producers (P_i ; i = 1:5) and consumers (C_j ; j = 1:5), positive interactions (indicated in green) are more likely to occur, because excreted metabolites (excreted by the producers) are consumed by the consumer species. Among producers or among consumers, the interactions are predominantly negative (indicated in red) as these species are more likely to compete for similar resources. (C) An example of a structured network with interaction hubs and its corresponding interaction matrix. The hub-species network contains species with unusually high numbers of ecological interactions compared to other species in the network. This can occur when some species perform a central role in the microbial ecosystem, for example when a hub-species produces a metabolite that is required for growth by many other species.
