# Supplementary material for: Species abundance correlations carry limited information about microbial network interactions
Source: PLoS Comput Biol. 2022 Sep 9;18(9):e1010491. doi: 10.1371/journal.pcbi.1010491 (PMC9518925; doi:10.1371/journal.pcbi.1010491)
Supplement: S5 Fig — (PDF) [file pcbi.1010491.s006.pdf]

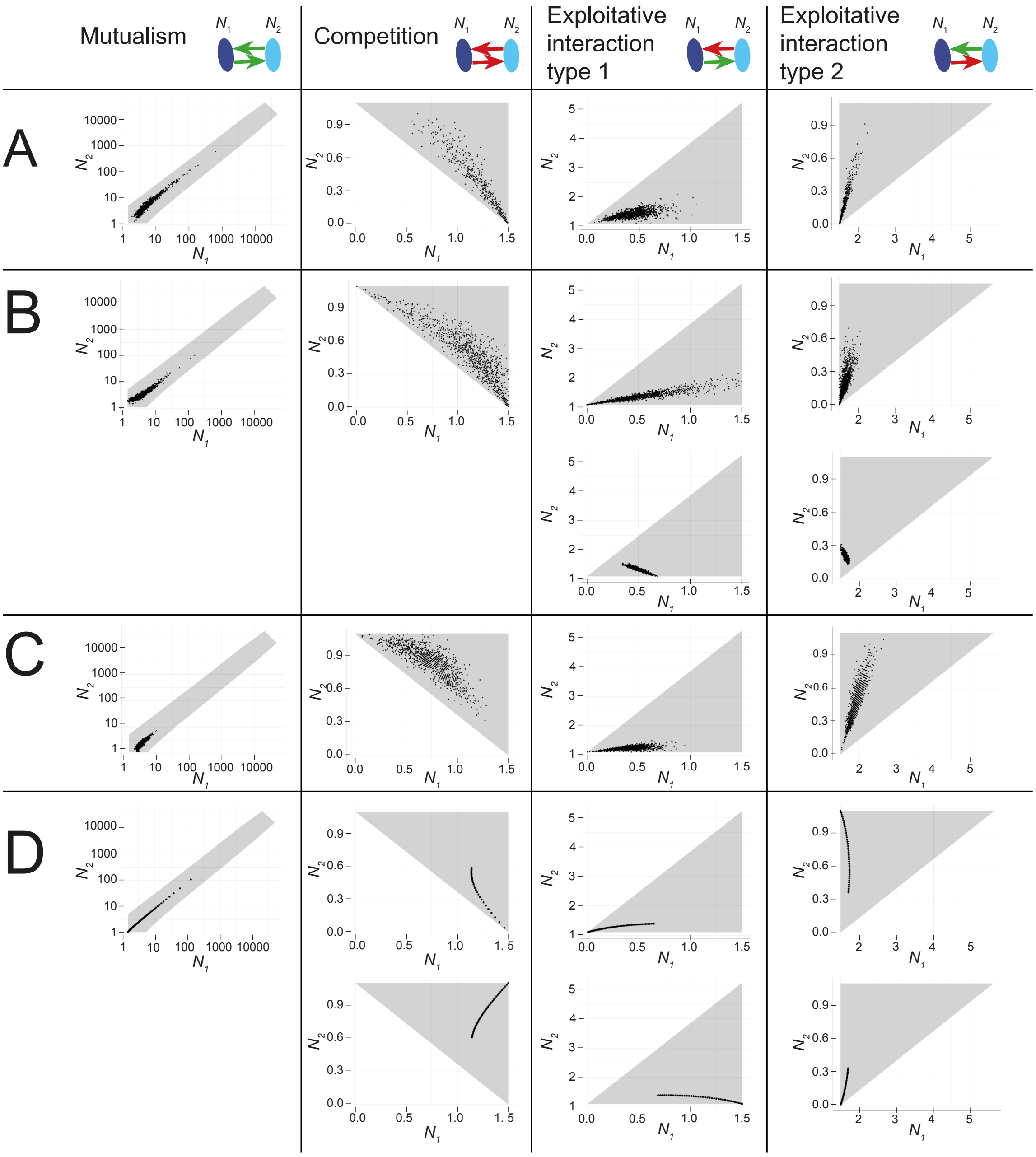

S5 Fig. The effect of α_{ij} on the correlations between the abundances of two bacterial species for different interactions mechanisms. The two species can co-exist under certain combinations of α_{ij} (S1 Text). The abundances of the two species N_1 and N_2 at equilibrium are shown as scatterplots and have been obtained by running the two-species Lotka-Volterra model with K_1 = 1.5; K_2 = 1.1; r_1 = 1; r_2 = 2 and α_{ij} drawn randomly from normal distributions with different combinations of means and standard deviations. In (A) the two distributions have different means and standard deviations: α_{12} ~ N(|0.5|, 0.1) and α_{21} ~ N(|0.7|, 0.2). In (B) the distributions have identical means, but different standard deviations: α_{12} ~ N(|0.5|, 0.2) and α_{21} ~ N(|0.5|, 0.1). For exploitative interactions we also show the situations that negative correlations can occur when the exploitative benefit displays much more variation than the harm to the other species, i.e. α_{12} ~ N(–0.5, 0.01) and α_{21} ~ N(0.5,

0.2) for exploitative interaction type 1, and α_{12} ~ N(0.5, 0.2) and α_{21} ~ N(–0.5, 0.01) for exploitative interaction type 2. In (C) interactions are randomly drawn from distributions with different means and identical standard deviations: α_{12} ~ N(|0.6|, 0.1) and α_{21} ~ N(|0.3|, 0.1). In (D) the interactions have identical strengths for the two species, namely $|\alpha_{12}| = |\alpha_{21}|$. The mutualistic interactions are drawn from the distribution $\alpha_{12} = \alpha_{21}$ ~ U(0, 2.5), for competition and exploitative interactions we show two different scenarios, namely $|\alpha_{12}| = |\alpha_{21}|$ ~ U(|0.4|, |2.5|) (upper graph) and $|\alpha_{12}| = |\alpha_{21}|$ ~ U(0, |0.4|) (lower graph). Because the two species have different carrying capacities, the two situations of exploitative interactions are different. The grey polygon indicates the area where co-existence is possible. Note that the axes ranges are different in each subplot.
