# Supplementary material for: Species abundance correlations carry limited information about microbial network interactions
Source: PLoS Comput Biol. 2022 Sep 9;18(9):e1010491. doi: 10.1371/journal.pcbi.1010491 (PMC9518925; doi:10.1371/journal.pcbi.1010491)
Supplement: S1 Table — (PDF) [file pcbi.1010491.s007.pdf]

S1 Table. Mann-Whitney U test results for the F1-scores of the base case model and for the F1-scores of the model with different sources of process variability. The significant values are indicated in bold.

**Scenario**	**p-value**
Variation in all parameters	**p-value < 0.001**
Variation in carrying capacities	**p-value < 0.001**
Variation in growth rates	**p-value < 0.001**
No variation in parameters	**p-value < 0.001**
Low measurement noise	**p-value < 0.001**
High measurement noise	**p-value < 0.001**
Low process noise	**p-value < 0.001**
High process noise	**p-value < 0.001**
Uniform distribution	p-value > 0.05
Unimodal distribution	**p-value < 0.001**
30 species system	**p-value < 0.001**
Producer-consumer network	**p-value < 0.001**
Hub-species network	**P-value < 0.001**
Mediate strict inference	p-value > 0.05
Strict inference	**p-value < 0.001**
Base case (3000 hosts)	**p-value < 0.001**
Producer consumer network (3000 hosts)	**p-value < 0.001**
Hub species network (3000 hosts)	**p-value < 0.001**
