# Supplementary material for: Species abundance correlations carry limited information about microbial network interactions
Source: PLoS Comput Biol. 2022 Sep 9;18(9):e1010491. doi: 10.1371/journal.pcbi.1010491 (PMC9518925; doi:10.1371/journal.pcbi.1010491)
Supplement: S2 Table — (PDF) [file pcbi.1010491.s008.pdf]

S2 Table. Mann-Whitney U test results for the F1-scores of the samples taken outside equilibrium relative to those taken at equilibrium (t_5 in Fig 5) and for the F1-scores of the samples taken outside equilibrium. The significant values are indicated in bold.

Variation in interactions	
**Timepoint**	**p-value**
Random	**p-value < 0.001**
t_1	**p-value < 0.001**
t_2	**p-value < 0.001**
t_3	**p-value < 0.001**
t_4	**p-value < 0.001**
Variation in carrying capacities	
**Timepoint**	**p-value**
Random	p-value > 0.05
t_1	**p-value < 0.001**
t_2	**p-value < 0.001**
t_3	**p-value < 0.001**
t_4	**p-value < 0.001**
Variation in growth rates	
**Timepoint**	**p-value**
Random	**p-value < 0.001**
t_1	**p-value < 0.001**
t_2	**p-value < 0.001**
t_3	**p-value < 0.001**
t_4	**p-value < 0.001**
