# Supplementary material for: Species abundance correlations carry limited information about microbial network interactions
Source: PLoS Comput Biol. 2022 Sep 9;18(9):e1010491. doi: 10.1371/journal.pcbi.1010491 (PMC9518925; doi:10.1371/journal.pcbi.1010491)
Supplement: Attachment — Submitted filename: Review PCOMPBIOL-D-22-00053.docx [file pcbi.1010491.s009.docx]

This paper is an important contribution for understanding the extent to which correlation analysis and co-occurrence networks are truly indicative of the microbial interaction landscape. It is clearly written and structured. This paper poses some valuable and tangible limitations to this method of parameter inference, providing important context to studies that have applied this method to inform their generalised Lotka-Volterra-type models, and I endorse publication of the work.

I have a few suggestions for improvement of the manuscript. Overall, the manuscript may benefit from questioning the nature of gLV-type models (and ecological networks overall) in their reliance on pairwise interactions before the Discussion section. A more explicit discussion of the role of higher-order interactions, and what this means for parameter inference, in the introduction section could thus be valuable as well.

**Abstract:**

Line 36: “In addition, we found that competitive and parasitic interactions can induce positive as well as negative correlations.” I believe (the meaning of) this sentence is not immediately intuitive for the new reader, and perhaps could thus benefit from rephrasing/clarification.

**Introduction:**

Line 72: “Negative interactions take place when toxic compounds produced by one species harm other bacteria” What about exploitative/trophic/parasitic (-/+ or -/+) interactions? Perhaps good to clarify the various forms of antagonistic interactions besides those that are mentioned (those mediated by competition and toxic metabolites). If, however, by this sentence is meant: negative interactions mediated by externally secreted compounds, it might be good to clarify this.

Line 79: “A common way to infer microbial networks from such cross-sectional data is by quantifying co-occurrence, e.g., through (partial) correlations, between bacterial phylogenetic groups.” I believe this sentence, and the introduction as a whole, would benefit from highlighting some studies that have done this and have derived important hypotheses/conclusions from this. Highlighting these studies – on top of ref [12] later on and the cited refs that have provided (network) tools or criticized these tools – can serve to drive home the importance of this paper in questioning the underlying meaning of correlation in mapping interaction landscapes altogether. Some papers that might be useful/relevant to cite here:

Chaffron, S., et al., *A global network of coexisting microbes from environmental and whole-genome sequence data.* Genome Res, 2010. **20**(7): p. 947-59

Stein, R.R., et al., *Ecological Modeling from Time-Series Inference: Insight into Dynamics and Stability of Intestinal Microbiota.* Plos Computational Biology, 2013. **9**(12)

Berry, D. and S. Widder, *Deciphering microbial interactions and detecting keystone species with co-occurrence networks.* Frontiers in Microbiology, 2014. **5**.

**Parameterization of the base case simulations:**

Line 175: “in the base case, density was 1⁄4 meaning that 3 out of 4 possible interactions were set to zero.” What justifies the use of this density? This section could benefit from referencing some studies about the prevalence of neutral interactions / limits of connectivity in microbial communities for instance.

Line 177 (and line 254): I believe Gaussian should be capitalised.

Overall, the types of distributions used to pull parameters from are explicitly mentioned, but not always justified. Perhaps the manuscript could benefit from having the mentioned distributions (normal, lognormal, uniform, unimodal, etc.) justified.

Line 192: “Note that, for the sake of simplicity, both the presence and the sign of interspecific interactions are kept constant across hosts.” This is a fair statement, however, it might be good to also explicitly state that this simplification is demonstrably untrue for many microbes in real communities, as they switch from interaction types while changing metabolic pathways for instance (this is touched upon in the Discussion, but could be good to shortly mention here as well).

**Discussion:**

L499: “Therefore, a correlation between the abundance of two species does not imply that those species are interacting [34]. Many of the detected correlations may be caused by shared environmental preferences rather than species interactions [35].” Also, co-occurring species may appear to be dependent on each other, while their co-occurrence can be explained by them actually sharing a similar dependency on a third party – so that co-occurrence, and hence apparent dependencies drawn from that, may also be explained by higher-order interactions (invisible to this method). Could this section benefit from mentioning this as well?

Overall, I believe the importance of the statement that this work underlines from L573, namely: “correlation-based methods are particularly insensitive for the detection of asymmetric interactions (such as parasitism, amensalism or commensalism), as direction of interaction cannot be recovered from co-occurrence data,” could be strengthened by once more emphasising the prevalence of such asymmetric interactions in microbial communities.
