# Supplementary material for: Species abundance correlations carry limited information about microbial network interactions
Source: PLoS Comput Biol. 2022 Sep 9;18(9):e1010491. doi: 10.1371/journal.pcbi.1010491 (PMC9518925; doi:10.1371/journal.pcbi.1010491)
Supplement: Attachment — Submitted filename: Rebuttal_PCOMPBIOL_26042022.docx [file pcbi.1010491.s010.docx]

Dr. Kiran Raosaheb Patil

Deputy Editor

*PLOS Computational Biology*

Leiden University Medical Center

Department of Biomedical Data Sciences

Einthovenweg 20

2333 ZC Leiden, The Netherlands

+31 6344 45 891

s.pinto@lumc.nl

Leiden, 26 April 2022

PCOMPBIOL-D-22-00053

Dear editors, dear Dr. Patil,

Thank you for giving us the opportunity to revise and resubmit our manuscript entitled “Species abundance correlations carry limited information about microbial network interactions” (previous title: “Species abundance correlations cannot distinguish interaction types in microbial networks”). The change in title was suggested by one of the reviewers' comments. In addition, we have also modified the abstract to better reflect the new title.

The reviewers’ comments have been most helpful in clarifying and refining our manuscript. We addressed all points to the best of our abilities, and we think the revised manuscript has improved because of the changes we have made. This holds especially for the part in which we present variations on the base case, where we added extra analyses and the more nuanced discussion of strengths and weaknesses of our model, as requested by both reviewers. We also thank the second reviewer for supplying useful additional references. Specific changes related to all the comments can be found after each respective comment.

Below is our point-by-point response to the reviewers’ comments. We report the changes in the main text or supplementary material that apply to each comment, with line numbers referring to the version without tracked changes.

On behalf of all co-authors, I thank you for considering our revised manuscript and hope you will find it to be suitable for publication in PLOS Computational Biology.

Also on behalf of the co-authors,

Yours sincerely,

Susanne Pinto, MSc

***********************

**Reviewer's Responses**

Comments to the Authors:

**Reviewer #1**

(1) Reviewer’s comment

In this manuscript, the authors approach the question of whether cross-sectional sampling of microbial communities, followed by correlation analysis of quantitative abundances, can reveal insights into the types of ecological interactions occurring between microbes in a community. The authors test this by performing Lotka-Volterra simulations under known interaction topologies, and varying the parameters and/or noise-levels during each run. They then perform partial correlation analysis across multiple snapshots that have resulted from the simulations, testing whether the original input interactions can be retrieved. Overall, the study is dealing with a relevant problem, and it is technically well executed.

(1) Response to reviewer

*We thank the reviewer for this assessment.*

(2) Reviewer’s comment

However, I doubt whether its findings are all that surprising/novel. Most scientists would doubt that detailed mechanistic (ecological) insights could ever be drawn from cross-sectional correlation analysis alone, and I am not aware of studies that conclude about fine differences between directionalities and types of interactions from mere correlation data.

(2) Response to reviewer

*We agree with the reviewer that some of the more theoretical oriented scientists could find the findings intuitive. However, in Faust & Raes (2012). Microbial interactions: from networks to models. [Nature Reviews Microbiology, 10, 538-550] it is suggested that ecological networks can be reconstructed from co-occurrence data. This paper has had a large influence in the microbiome field; at the moment it has been cited 1724 times according to scopus (2024 acc. Google scholar). Also, empirical studies that base their conclusions on cross-sectional correlations keep coming up, see among others Riera and Baldo Animal Microbiome (2020) and Vemuri et al. Nutrients (2022). Although directionality and types of interaction are not specifically defined in these microbiome studies, edges in co-occurrence networks are explicitly referred to as ecological interactions. Apparently, there is limited critical attitude towards such interpretation.*

*In our opinion, correlation networks have not been systematically investigated against a ground truth. In this study we address the question whether it is possible to draw ecological conclusions based on correlations, and secondly, how the results from correlation analyses can be interpreted. We show that correlation analysis can provide some insights on the underlying ecological network, in that the sign of the correlation found mostly agreed with the strongest pairwise interaction in our simulations. As such, we provide a counterpoint to the more negative criticism voiced before, e.g. by Carr et al. ISME Journal (2019). Moreover, we do present some counter-intuitive findings, for example the result that negative symmetric interactions can result in positive correlations and the finding that disturbances can improve the inference of interactions from cross-sectional data.*

(3) Reviewer’s comment

More importantly, there are more advanced techniques such as Structural Equation Models or Bayesian Networks, which attempt to infer causality from cross-sectional community data, but are not tested by the authors.

(3) Response to reviewer

*It is true that there are other methods, such as Structural Equation Models (SEM) and Bayesian networks, that might be superior to analyze cross-sectional data than correlations, for example in finding conditional independence or higher-order interactions. Indeed, we did not test these methods. It may thus seem that our ambitions with this manuscript are limited. However, seeing the omnipresence of correlation analysis in the microbiome field (Faust & Raes 2012; Berry and Widder 2014; Carr et al. 2019), we aimed to first address its performance rather than that of more complex methods. Besides, underlying these more advanced methods, there is still some kind of association technique, meaning that some of the findings from this research may also apply to future research on more advanced methods.*

*Another reason why we chose not to investigate methods concerned with causality, is that these often assume that networks are uni-directed and acyclic. This is highly unlikely in microbial ecology and it is debatable whether these are applicable to gut-microbiome data. In addition, methods like SEM require prior knowledge on the network structure where little is known about the network of microbial interactions. To our opinion testing these methods imply another research design and this was beyond the scope of this study.*

*We added a few sentences to the discussion to mention the pros and cons of more advanced methods applied to the microbiome field (new text highlighted in* ***bold****).*

*Revised version:*

*[line 589] “Furthermore, the interactions between bacteria might be much more complex than the additive and pairwise interactions that the gLV model assumes. Momeni et al. (2017) claimed that pairwise modeling will often fail to predict microbial dynamics, as many interactions occur through chemical production pathways (such as cross-feeding and nutrient competition) involving more than two species [38].* ***Correlation analysis fails to capture the resulting higher-order interactions, for which more advanced techniques, e.g. graphical models (Højsgaard et al., 2012), might be more appropriate. However, these techniques are also unable to recover directionality and type of interactions. Often they require more prior knowledge of the network of microbial interactions, time series or more fine-grained data on the pathways of interaction. More classical methods of separating direct from indirect interactions, e.g. path analysis [Wootton, 1994], rely on testing of specific alternative causal hypothesis, which can only be considered as a next step in network inference. To shed more light on causal pathways****, there is a need in microbial ecology for models that can describe the full set of metabolite concentrations, metabolic fluxes and species abundances within a community [39]. Metabolic modeling is a rapidly developing field, but it is still challenging, heavily based on a priori assumptions and ignoring fundamental aspects of ecology (like environmental changes) [40].”*

(4) Reviewer’s comment

Furthermore, one of their novel findings (namely that parasitic interactions can manifest in both positive as well as negative correlations) is perhaps somewhat unrealistic: negative correlations are only observed in setups where the positive effect of the host on the parasite is much smaller than the negative effect of the parasite on the host (cf. line 329 and following). This appears to be biologically unrealistic; most parasites have evolved to avoid overly straining the host … the above setup is somewhat difficult to perceive as biologically meaningful/stable.

(4) Response to reviewer

*We thank the reviewer for pointing out this lack of clarity. All interactions in the interaction matrix A are among bacteria. We did not model a parasitic effect of bacteria on the host. Parasitic interactions among bacteria can for example occur when a bacterial species excretes metabolites, which function as an antimicrobial to other bacteria in the direct surroundings. The negative effect on these other bacteria has a positive effect on the metabolites-producing bacterial species itself, because it increases the space and food availability for itself (Woyke et al. 2006).*

*To make this clearer, we adjusted the text (new text in* ***bold****)*

*Revised version:*

*[line 367] “Correlations under parasitic interactions* ***among bacteria****, benefitting one but harming the other species, generally yielded positive correlations …”*

*[line 396] “For parasitic interactions* ***among bacteria****, either a positive or negative correlation coefficient ρ could be found, …”*

(5) Reviewer’s comment

Also, given that generic interaction types (positive and negative) are by-and-large well detected (Fig. 2 and 3A), the title of the study is perhaps unnecessarily pessimistic. A bit more nuance would perhaps be more helpful to the reader.

(5) Response to reviewer

*We thank the reviewer for pointing out this positive aspect in the findings from our research. However, we also agree with the reviewer’s second comment that detailed mechanistic interpretations cannot be drawn from cross-sectional correlations alone. As pointed out in our response to this comment, we would like to counsel scientists working in the microbiome field that careful interpretation and validation is required when inferring networks from cross-sectional abundance data. We therefore adjusted the title to convey a more optimistic, yet tentative message. Instead of focusing on the daunting task of distinguishing between interaction types (e.g. between mutualism vs. commensalism in positive interactions), we now highlight the finding that some interaction types can be detected (e.g. pairwise interactions wherein the net effect on population growth is positive mostly appear as positive correlations).*

*Previous version:*

*“Species abundance correlations cannot distinguish interaction types in microbial networks”*

*Revised version:*

*“Species abundance correlations carry limited information about microbial network interactions”*

*We also rewrote our abstract to highlight our positive findings:*

*Previous version:*

*“Metagenomics-based studies on communities of microorganisms are mostly limited to only a few samples in time, presenting mere ‘snapshots’ of the microbial ecosystem. Inferring interspecific interactions from such cross-sectional data is a daunting task and likely prone to bias, as the ‘true’ networks governing microbiome dynamics are often unknown. Common methods to infer microbial interactions are based on co-occurrence measures, such as correlations between bacterial phylogenetic groups. We tested whether such methods are useful tools in inferring interaction networks. For this purpose, we simulated bacterial communities by means of the generalized Lotka-Volterra model, with variation in model parameters representing variability among hosts. Our results show that correlations can be indicative for presence of bacterial interactions, but only when measurement noise is low relative to the variation in interaction strengths between hosts. Indication of interaction was affected by process noise or sampling under non-equilibrium conditions in simulations. However, direction of interaction could not be recovered, making correlations unsuitable for detection of asymmetric interaction types. In addition, we found that competitive and parasitic interactions can induce positive as well as negative correlations. To summarize, correlations cannot distinguish microbial interaction types, therefore careful interpretation and validation is required when inferring networks from cross-sectional abundance data.”*

*Revised version (new text in* ***bold****):*

*[line 23]* ***Unraveling the network of interactions in ecological communities is a daunting task.*** *Common methods to infer* ***interspecific*** *interactions* ***from cross-sectional data*** *are based on co-occurrence measures.* ***For instance, interactions in the human microbiome are often inferred from correlations between the abundance of*** *bacterial phylogenetic groups* ***across subjects****. We tested whether such* ***correlation-based*** *methods* ***are indeed reliable*** ***for*** *inferring interaction networks. For this purpose, we simulated bacterial communities by means of the generalized Lotka-Volterra model, with variation in model parameters representing variability among hosts. Our results show that correlations can be indicative for presence of bacterial interactions, but only when measurement noise is low relative to the variation in interaction strengths between hosts. Indication of interaction was affected* ***by type of interaction network, process noise and sampling under non-equilibrium conditions.*** ***The sign of a correlation mostly coincided with the nature of the strongest pairwise interaction, but this is not necessarily the case. For instance,*** *we found that competitive interactions* ***may result in*** *positive as well as negative correlations.* ***Thus, cross-sectional abundance data carry limited information about the underlying web of interactions. Correlations in abundance may hint at interactions but require independent validation.****”*

(6) Reviewer’s comment

The parameter and design choices during the modelling are fairly arbitrary: the interaction networks are small and have an entirely random topology – meaning that important features such as interaction-hubs and -modules are not considered.

(6) Response to reviewer

*We thank the reviewer for highlighting this point, which makes a valuable contribution to the manuscript. We already did include a variation to the base case with interaction modules, namely producer-consumer networks which are explained on page 11. However, we did not yet consider interaction-hubs. Therefore, we have added an extra variation to the base case to investigate the effect of interaction-hubs on the inference. We found that a network with interaction-hubs results in a significantly (p-value < 0.001) lower F1-score, due to a reduced recall, as compared to the base case model. The results of this analysis are added to the revised Figure 4E under the entry “Hub species network”, alongside the results for the 30-species system and the producer-consumer network.*

*We explained what we did in the methods section:*

*Revised version (all new text):*

*[line 277] “****Thirdly, we simulated a microbial network with interaction hubs, i.e. a network containing species with unusually high numbers of ecological interactions compared to other species in the network (S5 Fig) (Goh et al. 2002). Hub-species networks were created according to the Barabási-Albert model (Barabási, 2016) and implemented with the barabasi.game function from the igraph package (version 1.2.11). In the network-generating algorithm, interactions are distributed according to a mechanism of preferential attachment. Thus, species with interactions obtain a higher chance of getting more interactions, resulting in a few ‘hub-species’ with many interactions. We constructed two scale-free directed graphs (with power = 2), denoting “incoming” and “outgoing” interactions, and combined these to obtain a bidirected graph. Density was kept similar to the base case model (1/4).”***

*To illustrate the differences between the random interaction network used in the base case and the interaction network with interaction hubs or with interaction-modules in variations to the base case, we added a figure and more detailed explanation to supplementary figure S5.*

*We added to the results section the following text (new text in* ***bold****):*

*Revised version:*

*[line 446] “Fig 4E shows the results for different network types. Increasing the number of species from 10 to 30 had a significant negative effect on the success of the inference (S2 Table)****, which was mainly due to reduced precision****. Conversely, F1-scores were improved as compared to the base-case when assuming a producer-consumer based network (S5 Fig and S2 Table)****, on account of an improved recall****.* ***The inference in a network with interaction hubs (as explained in S5 Fig) was significantly worse than in a random network, which could be attributed to a somewhat reduced recall.”***

(7) Reviewer’s comment

The authors rarely motivate their design and parameter choices, which makes the interpretation of their observations difficult.

(7) Response to reviewer

*We based our simulations on the generalized Lotka-Volterra (gLV) model, because this model can modulate different types of interactions and it is commonly used in microbiome studies. We feel that this choice has been amply motivated and discussed, including limitations of the gLV modeling approach. However, the reviewer is right that our parameter choices are chosen somewhat arbitrarily. Base-case parameter values were chosen from the perspective of manageability and feasibility. For example, the density of the interaction matrix A was chosen such that the matrix is not too sparse (this would give very few interactions to estimate) or too dense (which makes it harder for species to co-exist). This also applies to the strength of the interactions; too weak interactions will not be picked up by the correlation analysis, but too strong interactions would cause species to outcompete each other. Likewise, the growth rates were chosen in such a way that all species would reach equilibrium at convenient simulation time.*

*We dispute that our findings would be difficult to interpret. For example, the finding that the amount of noise in a study affects the performance of network inference is a general notion, just as the finding that performance depends on strength and variability of interactions in the host population. Even so, what constitutes “high measurement noise” or “strong interactions” strongly depends on context, and we cannot provide quantitative guidance in this respect. In our defense, we did not set out to do so, as we employed a generic set-up that may be representative of microbial ecosystems, but does not describe any real-world system in particular.*

*We added a paragraph in the methods section to notify the reader that this study is not based on a real-world microbial system, and is meant to provide qualitative rather than quantitative results (new text in* ***bold****).*

*Revised version:*

*[line 182] “We started with a base case and we added step by step variation to this case.* ***Note that the base-case parametrization does not reflect any particular real-world system. Rather, parameters were chosen in such a way to facilitate computation and promote co-existence between species. Variations to the base-case parameters are shown later on, but also here, findings should be appreciated from a qualitative rather than quantitative viewpoint.*** *In the base case the number of bacteria equals ten.”*

(8) Reviewer’s comment

The level of noise in the “high” noise settings seems large – in my opinion it is not clear whether such levels of measurement noise prevail in practice.

(8) Response to reviewer

*The reviewer is likely right that the level of measurement noise in microbiome studies is nowadays well-controlled, especially with the ongoing developments in sequencing and processing techniques. However, the order of magnitude of the measurement noise should be judged against the equilibrium levels of bacterial abundance in our simulation study. Seeing that carrying capacities were drawn from a U(0, 1) distribution, with equal probability of increased or decreased abundance due to interspecific interactions, the “high” noise setting with |𝜐| ~ U(0, 0.1) would correspond to coefficients of variation in the order of 10%. This seems not too far-fetched. Likewise, the “high” noise setting still has smaller variability than that in process-related parameters used to create between-host heterogeneity in bacterial abundances (i.e. lognormal σ = 0.25 is approximately equal to 25% coefficient of variation). This finding motivated us to conclude satisfactory performance “when measurement noise is low relative to the variation in interaction strengths between hosts.” We added some nuance and guidance on this point to the discussion (new text in* ***bold****).*

*Revised version:*

*[line 572] “If differences in bacterial abundances between hosts are mainly due to measurement noise, their correlations are not informative of underlying interactions.* ***In our simulations, with relative standard deviation in process-related parameters between hosts of about 25%, inference performed well as long as measurement noise had coefficients of variation well below 10% of mean bacterial abundances.*** *Strikingly, the inference of interactions was even improved when process noise was added. More research is needed to delineate the extent to which correlation analyses can be confounded by latent environmental drivers of microbial population dynamics, and how strongly one should condition on environmental or host homogeneity.”*

(9) Reviewer’s comment

Their "strict inference" analysis (Fig. 4F) would be more fair, if in cases where the true interaction weights are asymmetric, but the correlation analysis yields the “sign” of the interaction with the larger weight, this would be counted as “true positive” predictions. As stated now, the task cannot be solved, since the intended sign is not specified.

(9) Response to reviewer

*We thank the reviewer for pointing out this bias in our “strict inference” rule and agree that correct inference of the strongest interaction between each pair of species would be more fair. We therefore took the opportunity to add this scenario to the results; there are added to the revised figure 4F under the entry “Inference of intended sign”. Its performance was much more favorable compared to “strict inference” and comparable to the base case, as the slight reduction in F1-score was not significant (S2 Table).*

*We added the following text to the methods section to explain this scenario under ‘Assessment of correlation-based network inference’ (new text in* ***bold****):*

*Revised version*

*[line 316] “For a correctly classified interaction, either one or both interactions in the upper and lower part of the A matrix must have the same sign as in the lower part of the partial correlation matrix. This can produce a bias, because asymmetric interactions can result in a true positive result for correspondence of the correlation coefficient (ρ) with either interaction.* ***For example, for parasitic interactions, both negative and positive correlations are classified as true positive results.*** *Therefore,* ***we tested the effect of this bias on the success of network inference by specifying the intended sign in correlation analysis, as the sign of the strongest interaction in each pair of species. Hence, for a parasitic interaction, only a positive or a negative correlation is correct, depending on the weights of the asymmetric interactions. Secondly,*** *we also tested the effect of this bias on the success of network inference by setting the rule that* ***the sign of*** *both interactions must be matched by the inferred correlation coefficient. Hence, only mutualism and competition can be inferred correctly, as amensalism, commensalism and parasitism are asymmetric.”*

*We added the following text to the Results (new text in* ***bold****):*

*Revised version*

*[line 454] “Note that problems* ***may*** *arise with asymmetric relationships.* ***When using the rule that pairwise correlations should match the strongest interaction between both species involved as the intended sign, we found only a slight non-significant reduction in F1-score as compared to the base case scenario (Fig 4F and S2 Table). Thus, pairwise interactions wherein the net effect on population growth is positive or negative are mostly picked up as such in correlation analysis. However,*** *under the rule that mutual interactions must both be reflected in the sign of the correlations, asymmetric interactions cannot be recovered as correlations are symmetric. We indeed found much lower F1-scores when detection of asymmetric interactions was no longer considered as a true positive result after inferring a significant correlation coefficient ρ (either positive or negative) (Fig 4F).“*

In addition to the concerns above, the reviewer raised a number of minor points, addressed as outlined below:

(10) Reviewer’s comment

“only a few samples at a time” repeatedly used, but misleading, since most studies have dozens or hundreds of samples (not to mention large scale projects with thousands)

(10) Response to reviewer

*In the human microbiome field, there are indeed some large studies, for example HMP (Turnbaugh et al. 2007) and Caporaso et al. (2011). However, as data collection and sequencing are still time consuming (and expensive), there is typically a trade-off in studies depending on human material, with either many samples derived from a few subjects or few samples derived from many subjects. Even so, the reviewer alerted us to the possibility that “only a few samples at a time” may be misread as representing cross-sectional studies involving only a few subjects. To avoid misinterpretation, we therefore added the following nuances to the text (new text in* ***bold****).*

*Introduction:*

*[line 79] “Most human microbiome studies are limited to only a few samples in time, presenting mere ‘snapshots’ of the microbial ecosystem****, even if these are derived from hundreds of human hosts****.”*

*The total number of hosts was fixed at 300 in all our simulations. To investigate dependence on sample size, we also added a scenario with 3000 hosts, for the base-case with random interaction networks as well as for the scenarios with structured (i.e. producer-consumer and hub-species) networks. The results have been added to the revised Figure 4G, and show that performance of network reconstruction generally improves when more hosts are sampled.*

*We added the following text to the methods section (all new text):*

*[line 289] “****Next, we also investigated how network inference is affected by sample size by considering a scenario with 3000 instead of 300 hosts. We did this for the base-case model with random interaction networks, as well as for the producer-consumer and hub-species networks described above.”***

*We added the following text to the Results (all new text):*

*[line 465] “****Finally, we verified that network inference improved with increasing sample size. This applied to models with random as well as structured interactions networks (Figure 4G). In the base case, precision was somewhat reduced at increased sample size notwithstanding Benjamini-Hochberg control. However, this was compensated by substantially improved recall, resulting in significantly increased F1-scores. Interestingly, precision stayed more or less constant at increased sample size in producer-consumer and hub-species networks, whereas recall improved but remained somewhat behind that of random networks.“***

(11) Reviewer’s comment

Figure reference in line 329 should probably be Fig. 2E/2F, since 2C/2D don’t show parasitism

(11) Response to reviewer

*This comment is correct, we thank the reviewer for pointing out this mistake, we have corrected this in the text.*

(12) Reviewer’s comment

L 407: the authors use the term significant without statistically quantifying this statement

(12) Response to reviewer

*Significance level in network inference is noted in the footnote to Table 1: “Only significant partial correlations (with p < 0.05) are considered after correction for multiple testing with Benjamini-Hochberg procedure.” We did not quantify all the p-values regarding model comparisons in the main text, but supplementary table S2 and S3 shows all p-values belonging to figure 4 and figure 5.*

(13) Reviewer’s comment

L 472: but only in very rare cases (see Fig. 3A), this should be explicitly mentioned here

(13) Response to reviewer

*We thank the reviewer for this assessment, we made a change to the text on page 21.*

*Revised version (new text in* ***bold****):*

*[line 530] “Symmetric interaction types, where species involved affect each other’s growth in a qualitatively similar way (competition, mutualism) can be recovered, although competitive interactions may also result in positive correlations****, albeit in very rare cases****.”*

(14) Reviewer’s comment

L 480-81: where do the authors demonstrate that omnipresence of species is generally required? If they do, this should be referred to here

(14) Response to reviewer

*We thank the reviewer for this comment and realized that the sentence it refers to was not correct. Omnipresence of species is not required, but in our model we assume that the topology of the network is the same in all interaction networks and that the differences between hosts arise because of differences in process-related parameters, such as interaction strength. We have reformulated this sentence to reflect this interpretation.*

*Revised version (new text in* ***bold****):*

*[line 538] “Still, as illustrated by our analysis, correlations in microbial abundance across independently sampled hosts can be indicative for underlying ecological interactions under host-specific variation in microbial population dynamics.* ***That is, if microbial groups of interest are omnipresent and their interactions are appropriately captured by generalized Lotka-Volterra (gLV) dynamics, the variation in population abundances should be driven by interindividual variability in population-dynamic parameters.”***

(15) Reviewer’s comment

L553: where do the authors show that positive interactions are more readily detectable than negative ones? (to the contrary, L359-60 reads: “There was no difference between the successful inference of positive interactions over negative interactions”)

(15) Response to reviewer

*We did indeed not observe a difference between the successful inference of positive interactions and negative interactions in figure 3. However, in the simulations underlying this illustration we did not consider symmetric interactions. In the first example (Figure 2) we showed that symmetric negative interactions can result in positive correlations, whereas the reverse was not observed. Hence, we conclude that positive interactions are generally better detectable than negative ones.*

*We made this distinction clearer in the results section:*

*Revised version (new text in* ***bold****):*

*[line 399] “****Contrary to the results that included symmetric interactions,*** *there was no difference between the successful inference of positive interactions over negative interactions in any interaction type (Fig 3B).“*

*In the Discussion, we made the correspondence between our findings and those of an empirical study more precise:*

*Revised version (new text in* ***bold****):*

*[line 627] “There are similarities in their findings to our results. For example, they found that weak interactions are missed more often than interactions above a certain threshold. They also concluded that the ability to correctly detect a true link varies across different interaction types****, and that positive interactions are better detected than negative interactions. Interestingly, in line with our results, they also found that negative interactions are misclassified as positive interactions more often than vice versa.****“*

(16) Reviewer’s comment

Fig. 1: may need re-arrangement (B comes before A)

(16) Response to reviewer

*We adjusted the figure and the figure legend. We now call the upper matrix A (in line with the interaction matrix specified in the text) and no longer name the network itself, as the whole analysis revolves around it.*

**Reviewer #2**

(17) Reviewer’s comment

This paper is an important contribution for understanding the extent to which correlation analysis and co-occurrence networks are truly indicative of the microbial interaction landscape. It is clearly written and structured. This paper poses some valuable and tangible limitations to this method of parameter inference, providing important context to studies that have applied this method to inform their generalised Lotka-Volterra-type models, and I endorse publication of the work.

(17) Response to reviewer

*We thank the reviewer for this assessment*

I have a few suggestions for improvement of the manuscript.

(18) Reviewer’s comment

Overall, the manuscript may benefit from questioning the nature of gLV-type models (and ecological networks overall) in their reliance on pairwise interactions before the Discussion section. A more explicit discussion of the role of higher-order interactions, and what this means for parameter inference, in the introduction section could thus be valuable as well.

(18) Response to reviewer

*It would be very interesting to investigate and evaluate more complex models and ecological networks, with for example higher-order interactions. However, we wanted to start with a modest objective, because we felt that the relation between pairwise correlations and pairwise ecological interactions first deserved investigation. Moreover, if correlations are failing to infer interactions from an ‘easy’ model, such as the generalized Lotka-Volterra model, it would be even harder for correlation analysis to enable inference of interactions from a more complex model.*

*To make our reasoning for using gLV-type models in the current study explicit, and to alert the reader that such models rely on pairwise interactions, we added the following sentences to the Introduction:*

*Revised version (new text in* ***bold****):*

*[line 110] “The model is commonly used in microbiome studies for different aims: to simulate microbial communities under various interaction structures [16], to infer interaction structure from time-series data [17], to forecast population dynamics after a perturbation [18], to infer the network topology from steady state samples [19] and to identify the efficiency of intervention protocols in altering the state of a system via the addition or subtraction of microbial species [20].* ***In ecology, gLV-type models have been questioned for their reliance on pairwise additive interactions, as well as for the strictly linear effects imposed on interspecific interactions. Nonetheless, from the perspective of network inference, it makes sense to first investigate gLV-type models, as their first-order description of ecological dependencies, specified through a pairwise interaction matrix, resembles the objective of correlation analysis and most network models (Faust & Raes, 2012).”***

*In addition, we added the following text to the discussion section to more explicitly address this point (new text in* ***bold****).*

*Revised version:*

*[line 589] “Furthermore, the interactions between bacteria might be much more complex than the additive and pairwise interactions that the gLV model assumes. Momeni et al (2017) claimed that pairwise modeling will often fail to predict microbial dynamics, as many interactions occur through chemical production pathways (such as cross-feeding and nutrient competition) involving more than two species [38].* ***Correlation analysis fails to capture the resulting higher-order interactions, for which more advanced techniques, e.g. graphical models (Højsgaard et al., 2012), might be more appropriate. However, these techniques are also unable to recover directionality and type of interactions. Doing so would require more prior knowledge of the network of microbial interactions, or more fine-grained data on the pathways of interaction. More classical methods of separating direct from indirect interactions, e.g. path analysis (Wootton, 1994), rely on testing of specific alternative causal hypothesis, which can only be considered as a next step in network inference. To shed more light on causal pathways****, there is a need in microbial ecology for models that can describe the full set of metabolite concentrations, metabolic fluxes and species abundances within a community [39]. Metabolic modeling is a rapidly developing field, but it is still challenging, heavily based on a priori assumptions and ignoring fundamental aspects of ecology (like environmental changes) [40].“*

Abstract:

(19) Reviewer’s comment

Line 36: “In addition, we found that competitive and parasitic interactions can induce positive as well as negative correlations.” I believe (the meaning of) this sentence is not immediately intuitive for the new reader, and perhaps could thus benefit from rephrasing/clarification.

(19) Response to reviewer

*We are thankful that the reviewer mentioned this non intuitive result and rewrote the sentence for clarification.*

*Revised version (new text in* ***bold****):*

*[line 33] “****The sign of a correlation mostly coincided with the nature of the strongest pairwise interaction, but this is not necessarily the case. For instance,*** *we found that competitive interactions* ***may result in*** *positive as well as negative correlations.”*

Introduction:

(20) Reviewer’s comment

Line 72: “Negative interactions take place when toxic compounds produced by one species harm other bacteria” What about exploitative/trophic/parasitic (-/+ or -/+) interactions? Perhaps good to clarify the various forms of antagonistic interactions besides those that are mentioned (those mediated by competition and toxic metabolites). If, however, by this sentence is meant: negative interactions mediated by externally secreted compounds, it might be good to clarify this.

(20) Response to reviewer

*We thank the reviewer for pointing out this lack of clarity. In this sentence we gave an example, but indeed not all interactions occur through this mechanism. Therefore, we changed this sentence as follows:*

*Revised version (new text in* ***bold****):*

*[line 71] “****Among other mechanisms, for example,*** *negative interactions take place when toxic compounds produced by one species harm other bacteria, whereas positive interactions occur when bacteria feed on the nutrients that are produced by others.* ***Besides, many different forms of interactions exist, depending on the effects experienced by the species involved.*** *Knowledge of interspecific interactions in the human microbiome is paramount to understand ecological processes and compositional changes in relation to health and disease [7, 8].”*

(21) Reviewer’s comment

Line 79: “A common way to infer microbial networks from such cross-sectional data is by quantifying co-occurrence, e.g., through (partial) correlations, between bacterial phylogenetic groups.” I believe this sentence, and the introduction as a whole, would benefit from highlighting some studies that have done this and have derived important hypotheses/conclusions from this. Highlighting these studies – on top of ref [12] later on and the cited refs that have provided (network) tools or criticized these tools – can serve to drive home the importance of this paper in questioning the underlying meaning of correlation in mapping interaction landscapes altogether. Some papers that might be useful/relevant to cite here:

Chaffron, S., et al., *A global network of coexisting microbes from environmental and whole-genome sequence data.* Genome Res, 2010. **20**(7): p. 947-59

Stein, R.R., et al., *Ecological Modeling from Time-Series Inference: Insight into Dynamics and Stability of Intestinal Microbiota.* Plos Computational Biology, 2013. **9**(12)

Berry, D. and S. Widder, *Deciphering microbial interactions and detecting keystone species with co-occurrence networks.* Frontiers in Microbiology, 2014. **5**.

(21) Response to reviewer

*We agree on the sparsity of references about studies that have derived conclusions based on cross-sectional correlation analyses. However, the references to Stein (2013) and to Berry & Widder (2014) already figured prominently in our manuscript. We thank the reviewer for supplying us with useful additional references. We added text to the introduction to highlight the fact these papers have made use of correlations as a proxy for interactions.*

*Revised version (new text in* ***bold****):*

*[line 81] “A common way to infer microbial networks from such cross-sectional data is by quantifying co-occurrence, e.g., through (partial) correlations, between bacterial phylogenetic groups.* ***Several different conclusions have been derived from such endeavors, for example on species associations that reflect shared or overlapping niche preferences (Chaffron et al., 2010), microbial community structure (Riera and Baldo, 2020; Vemuri et al., 2022), the resilience of microbial communities to perturbations (Stein et al., 2013) and keystone species in microbial networks (Berry and Widder, 2014).*** *Currently there are several correlation-based network tools available that can deal with the difficulties of microbiome data, such as the compositionality [9-11]. The potential of correlation-based approaches for uncovering microbial networks has been highlighted in previous research [12].”*

Parameterization of the base case simulations:

(22) Reviewer’s comment

Line 175: “in the base case, density was 1⁄4 meaning that 3 out of 4 possible interactions were set to zero.” What justifies the use of this density? This section could benefit from referencing some studies about the prevalence of neutral interactions / limits of connectivity in microbial communities for instance.

(22) Response to reviewer

*To our knowledge, there is no empirical evidence for the density of microbial networks in human microbiota. Therefore, our parameter choices are chosen somewhat arbitrarily and display a sweet spot between manageability and interest. A very sparse network would give very few interactions to estimate, but too dense interaction networks make it harder for species to co-exist.*

*We added a paragraph in the methods section to notify the reader that this study is not based on a real-world microbial system, and is meant to provide qualitative rather than quantitative results (new text in* ***bold****).*

*Revised version:*

*[line 182] “We started with a base case and we added step by step variation to this case.* ***Note that the base-case parametrization does not reflect any particular real-word system. Rather, parameters were chosen in such a way to facilitate computation and promote co-existence between species. Variation to the base-case parameters are shown later on, but also here findings should be appreciated from a qualitative than quantitative viewpoint.*** *In the base case the number of bacteria equals ten.”*

(23) Reviewer’s comment

Line 177 (and line 254): I believe Gaussian should be capitalised.

(23) Response to reviewer

*We agree with the reviewer, and changed this throughout the text.*

(24) Reviewer’s comment

Overall, the types of distributions used to pull parameters from are explicitly mentioned, but not always justified. Perhaps the manuscript could benefit from having the mentioned distributions (normal, lognormal, uniform, unimodal, etc.) justified.

(24) Response to reviewer

*As stated before, the ground truth for interactions in the human microbiome is not known. Therefore we used different variations (e.g. bimodal, uniform, normal), to show the reader the effect of different distributions with respect to interspecific interactions. Regarding the use of log-normal distributions, these were mainly employed to model fold-changes representing host variability around population means, while at the same time safeguard sign of interactions or process parameters. This is now mentioned in the methods section (new text in* ***bold****):*

*Revised version:*

*[line 208] “Note that, for the sake of simplicity,* ***the use of log-normal distributions was adopted to induce fold-changes around population means, where*** *both the presence and the sign of interspecific interactions are kept constant across hosts.”*

(25) Reviewer’s comment

Line 192: “Note that, for the sake of simplicity, both the presence and the sign of interspecific interactions are kept constant across hosts.” This is a fair statement, however, it might be good to also explicitly state that this simplification is demonstrably untrue for many microbes in real communities, as they switch from interaction types while changing metabolic pathways for instance (this is touched upon in the Discussion, but could be good to shortly mention here as well).

(25) Response to reviewer

*We thank the reviewer for this comment, we adjusted the text to show the nuance in this statement.*

*Revised version (new text in* ***bold****):*

*[line 208] “Note that, for the sake of simplicity,* ***the use of log-normal distributions was adopted to induce fold-changes around population means, where*** *both the presence and the sign of interspecific interactions are kept constant across hosts.* ***However, this may be untrue in real microbiota as many microbes can change metabolic pathways and therefore may switch from interaction types and interaction partners.*** *In the ...”*

Discussion:

(26) Reviewer’s comment

L499: “Therefore, a correlation between the abundance of two species does not imply that those species are interacting [34]. Many of the detected correlations may be caused by shared environmental preferences rather than species interactions [35].” Also, co-occurring species may appear to be dependent on each other, while their co-occurrence can be explained by them actually sharing a similar dependency on a third party – so that co-occurrence, and hence apparent dependencies drawn from that, may also be explained by higher-order interactions (invisible to this method). Could this section benefit from mentioning this as well?

(26) Response to reviewer

*We thank the reviewer for this nice addition. Indeed, bacteria can share metabolic functions or depend on a third party, that is not necessarily in the data (for example a virus, bacteriophage or fungus). In practice this is often true. Therefore, we added this to the discussion.*

*Revised version (new text in* ***bold****):*

*[line 562] “Therefore, a correlation between the abundance of two species does not imply that those species are interacting [34]. Many of the detected correlations may be caused by shared environmental preferences rather than species interactions [35]. Such kind of environmental filtering can mask putative between-species interactions as well as induce spurious correlations [13].* ***Also, co-occurring species may appear to be dependent on each other, while their co-occurrence can be explained by them actually sharing a similar dependency on a third party – so that co-occurrence, and hence apparent dependencies drawn from that, may also be explained by higher-order interactions (Mayfield & Stouffer, 2017) .”***

(27) Reviewer’s comment

Overall, I believe the importance of the statement that this work underlines from L573, namely: “correlation-based methods are particularly insensitive for the detection of asymmetric interactions (such as parasitism, amensalism or commensalism), as direction of interaction cannot be recovered from co-occurrence data,” could be strengthened by once more emphasising the prevalence of such asymmetric interactions in microbial communities.

(27) Response to reviewer

*We thank the reviewer for this comment; indeed, we show that asymmetric interaction types are more difficult to detect and infer correctly than pairwise interactions where both species involved experience similar effects. However, we do not know, nor claim to know, to what extent such asymmetric interactions are prevalent in microbial communities. We must deduce this knowledge from in vitro studies and from studies on other ecological systems (Budding et al., 2009), as in vivo studies on human microbiota are rare. We know from examples with toxic compounds that the benefit of one is often in disadvantage of the other (Hibbing et al, 2010), but the amount of parasitism is unclear. We therefore refrain from making such a statement, and leave it to others to anticipate the omnipresence of asymmetric interactions in real-world systems, and thereby the pitfalls of using correlation analysis in deciphering such interactions.*

**References**

Barabási AL. Network Science [1st ed]. Cambridge University Press. 2016: 1-475.

Berry D, Widder S. Deciphering microbial interactions and detecting keystone species with co-occurrence networks. Frontiers in Microbiology. 2014; 5: 1-14.

Budding AE, Ingham CJ, Bitter W, Vandenbroucke-Grauls CM, Schneeberger PM. The Dienes phenomenon: competition and territoriality in Swarming Proteus mirabilis. J Bacteriol. 2009;191(12):3892-900.

Caporaso JG, Lauber CL, Costello EK, Berg-Lyons D, Gonzalez A, Stombaugh J, et al. Moving pictures of the human microbiome. Genome biology. 2011;12(5):50.

Carr A, Diener C, Baliga NS, Gibbons SM. Use and abuse of correlation analyses in microbial ecology. The The International Society of Microbial Ecology Journal. 2019; 13: 2647–2655.

Chaffron S, Rehrauer H, Pernthaler J, von Mering C., A global network of coexisting microbes from environmental and whole-genome sequence data. Genome Res. 2010; 20(7): 947-59.

Faust K, Raes J. Microbial interactions: from networks to models. Nature Reviews Microbiology. 2012; 10: 538-550.

Goh K-Il, Oh E, Jeong H, Kahng B, Kim D. Classification of scale-free networks. PNAS. 2002; 99(20): 12583–12588.

Hibbing ME, Fuqua C, Parsek MR, Peterson SB. Bacterial competition: Surviving and thriving in the microbial jungle. Nature Reviews Microbiology. 2010; 8: 15-25.

Højsgaard S, Edwards D, Lauritzen S. Graphical models with R. Springer Science & Business Media. 2012.

Mayfield M, Stouffer D. Higher-order interactions capture unexplained complexity in diverse communities. Nat Ecol Evol. 2017; 1: 0062.

Riera JL, Baldo L. Animal Microbial co-occurrence networks of gut microbiota reveal community conservation and diet-associated shifts in cichlid fishes. Microbiome. 2020; 2: 36.

Stein RR, Bucci V, Toussaint NC, Buffie CG, Rätsch G, Pamer EG, et al. Ecological modeling from time-series inference: insight into dynamics and stability of intestinal microbiota. Plos Computational Biology. 2013; 9: 1-11.

Turnbaugh P, Ley R, Hamady M, Fraser-Liggett CM, Knight R, Gordon JI. The Human Microbiome Project. Nature. 2007; 449: 804–810.

Vemuri R, Martoni CJ, Kavanagh K, Eri R. Lactobacillus acidophilus DDS-1 Modulates the Gut

Microbial Co-Occurrence Networks in Aging Mice. Nutrients. 2022; 14: 977.

Woyke T, Teeling H, Ivanova NN, Huntemann M, Richter M, Gloeckner FO, et al. Symbiosis insights through metagenomic analysis of a microbial consortium. Nature. 2006; 443(7114): 950-955.

Wootton JT. Predicting Direct and Indirect Effects: An Integrated Approach Using Experiments and Path Analysis. Ecology. 1994; 75(1): 151-165.

***********************

**Have the authors made all data and (if applicable) computational code underlying the findings in their manuscript fully available?**
The [PLOS Data policy](https://journals.plos.org/ploscompbiol/s/materials-and-software-sharing) requires authors to make all data and code underlying the findings described in their manuscript fully available without restriction, with rare exception (please refer to the Data Availability Statement in the manuscript PDF file). The data and code should be provided as part of the manuscript or its supporting information, or deposited to a public repository. For example, in addition to summary statistics, the data points behind means, medians and variance measures should be available. If there are restrictions on publicly sharing data or code —e.g. participant privacy or use of data from a third party—those must be specified.

Reviewer #1: Yes

Reviewer #2: Yes

PLOS authors have the option to publish the peer review history of their article ([what does this mean?](https://journals.plos.org/ploscompbiol/s/editorial-and-peer-review-process#loc-peer-review-history)). If published, this will include your full peer review and any attached files.


If you choose “no”, your identity will remain anonymous but your review may still be made public.


**Do you want your identity to be public for this peer review?** For information about this choice, including consent withdrawal, please see our [Privacy Policy](https://www.plos.org/privacy-policy).

Reviewer #1: No

Reviewer #2: No

Figure Files:

While revising your submission, please upload your figure files to the Preflight Analysis and Conversion Engine (PACE) digital diagnostic tool, [https://pacev2.apexcovantage.com](https://pacev2.apexcovantage.com/). PACE helps ensure that figures meet PLOS requirements. To use PACE, you must first register as a user. Then, login and navigate to the UPLOAD tab, where you will find detailed instructions on how to use the tool. If you encounter any issues or have any questions when using PACE, please email us at [figures@plos.org](mailto:figures@plos.org).

Data Requirements:

Please note that, as a condition of publication, PLOS' data policy requires that you make available all data used to draw the conclusions outlined in your manuscript. Data must be deposited in an appropriate repository, included within the body of the manuscript, or uploaded as supporting information. This includes all numerical values that were used to generate graphs, histograms etc.. For an example in PLOS Biology see here: http://www.plosbiology.org/article/info%3Adoi%2F10.1371%2Fjournal.pbio.1001908#s5.

Reproducibility:

To enhance the reproducibility of your results, we recommend that you deposit your laboratory protocols in protocols.io, where a protocol can be assigned its own identifier (DOI) such that it can be cited independently in the future. Additionally, PLOS ONE offers an option to publish peer-reviewed clinical study protocols. Read more information on sharing protocols at <https://plos.org/protocols?utm_medium=editorial-email&utm_source=authorletters&utm_campaign=protocols>
