# Supplementary material for: Species abundance correlations carry limited information about microbial network interactions
Source: PLoS Comput Biol. 2022 Sep 9;18(9):e1010491. doi: 10.1371/journal.pcbi.1010491 (PMC9518925; doi:10.1371/journal.pcbi.1010491)
Supplement: Attachment — Submitted filename: Rebuttal_PCOMPBIOL_10072022.docx [file pcbi.1010491.s011.docx]

Dr. Kiran Raosaheb Patil

Deputy Editor

*PLOS Computational Biology*

Leiden University Medical Center

Department of Biomedical Data Sciences

Einthovenweg 20

2333 ZC Leiden, The Netherlands

+31 6344 45 891

s.pinto@lumc.nl

Leiden, 12 July 2022

PCOMPBIOL-D-22-00053R2

Dear editors, dear Dr. Patil,

Thank you for giving us the opportunity to revise and resubmit our manuscript entitled “Species abundance correlations carry limited information about microbial network interactions”. All comments have been most helpful in improving our manuscript. This holds especially for the introduction and discussion, where we have added the suggestions for clarification and distinction, with reference to the suggested literature.

Below is our point-by-point response to the comments raised by the editor and reviewer. We report the changes in the main text that apply to each comment, with line numbers referring to the version without tracked changes.

On behalf of all co-authors, I thank you for considering our revised manuscript and hope you will find it to be suitable for publication in PLOS Computational Biology.

Also on behalf of the co-authors,

Yours sincerely,

Susanne Pinto, MSc

***********************

**Editors Response**

Comment to the Authors:

(1) Editor’s comment

I would recommend to shape the Introduction and Discussion to reflect extensive studies that have rather successfully used metabolic models in conjunction with co-occurrence analyses (binary as well as higher-order): e.g. Freilich et al. Nat Communications, 2011 and Machado et al. Nat Ecol Evol, 2021. The results from studies could be used to balance your discussion around the limits / benefits of correlational analyses.

(1) Response to the editor

*We agree that the introduction and discussion could have been more positive towards incorporation of findings from co-occurrence analyses in metabolic models, and we thank the editor for providing us with the useful suggestions of the literature. We added the following sentences to the introduction:*

*[line 100] “****Apart from*** *the challenges of metagenomic-based abundance data and disagreement between various network tools, here we question whether correlations itself are at all useful to distinguish between different ecological interaction types.* ***Resource competition and metabolic cooperation have been successfully inferred within environmental microbiomes, by linking ecological distribution data to multi-species metabolic models and subsequent verification of putative interactions by means of experimental co-growth analysis (Freilich et al. 2011). However, host-associated microbiomes often include non-culturable organisms, without information on nutrient requirements or metabolic function. Likewise, performance of correlation analysis in relation to alternative interaction types in human microbiota*** *is not well understood and deserves further investigation.”*

*and the discussion:*

*[line 623] “To shed more light on causal pathways, there is a need in microbial ecology for models that can describe the full set of metabolite concentrations, metabolic fluxes and species abundances within a community [47].* ***Based on metabolic modelling, Freilich et al. (2011) concluded that cooperative interactions are relatively rare among free-living bacteria and, if present, are often unidirectional. In contrast, Machado et al. (2021) suggested that mutualistic interactions are much more common among host-associated bacteria, that often form highly cooperative communities and have smaller genomes and fewer metabolic genes compared to other species. Cooperative communities are resilient to nutrient change and adaptable to a wide variety of different environments, including the human body (Freilich et al. 2011; Machado et al. 2021). Metabolic modeling is still challenging and heavily based on a priori assumptions, but is also a rapidly developing field that may prove useful for computational validation of correlation-based interaction networks*** *(Succurro et al., 2018).”*

**Reviewer's Responses**

Comments to the Authors:

(2) Reviewer’s comment

The authors have improved, extended and clarified the manuscript. There are a few (minor) issues left, which if addressed could further improve the manuscript.

(2) Response to reviewer

*We thank the reviewer for the assessment and feedback.*

(3) Reviewer’s comment

with regard to author response 2: “have not been systematically investigated against a ground truth” ... just for clarification: using mathematical models (gLV and others) as a ground truth has been done extensively in the literature (e.g. Weiss et al. (2016), also cited in the manuscript), albeit in variations that differ from what the authors did.

(3) Response to reviewer

*The reviewer is correct in stating that mathematical models have been used as ground truth, but in variations that differ from what we did. To stress that our work differs from previous endeavours in this regard, we have added the following sentence to the Introduction:*

*[line 111] “Correspondence of correlations with ecological interactions needs to be studied against a known ground truth, which can be achieved by means of simulation.* ***Mathematical models have been used as ground truth in assessment of correlation network techniques before (e.g. Weiss et al. 2016), but correlation networks have not been systematically investigated against distinct interaction types in dynamic models. This requires elucidation*** *especially as the ‘true’ ecological networks governing microbiome dynamics are still unknown.”*

(4) Reviewer’s comment

With regard to author response 3: “However, these techniques are also unable to recover directionality and type of interactions” ... SEMs/Bayesian networks do infer directed graphs (albeit with limitations), this sentence therefore seems misleading in the current form and requires either relevant references or proper explanation (similar to the response provided by the authors). Suggestion: “It is unclear, how well directed links predicted by these methods recover true ecological interaction types.”

(4) Response to reviewer

*We concur with the reviewer that the specific sentence may be misinterpreted, and we agree with the nuanced statement suggested instead. We have inserted this sentence in the Discussion:*

*[line 615] “Correlation analysis fails to capture the resulting higher-order interactions, for which more advanced techniques, e.g. graphical models (Højsgaard, et al., 2012), might be more appropriate****. It is unclear, how well directed links predicted by these methods recover true ecological interaction types. Often,*** *they require more prior knowledge of the network of microbial interactions, time series or more fine-grained data on the pathways of interaction.* ***Moreover, microbial networks can be bi-directed and cyclic (Freilich et al. 2011), which poses problems for inference of directionality and type of interactions from mere cross-sectional data.*** *More classical methods of separating direct from indirect interactions, e.g. path analysis (Wootton, 1994), rely on testing of specific alternative causal hypotheses, which can only be considered as a next step in network inference.”*

(5) Reviewer’s comment

with regard to author response 4: For clarification ... the given example constitutes not parasitism, but interference competition, where one species actively prevents another from obtaining resources used by both. The resulting interaction pattern would not be +/-, but rather -(weak)/-(strong), with one strong and one weak competitor. The original point stands: relationships in which one species simultaneously benefits from and harms another species are more likely to be stable if the exploitative benefit for species 1 outweighs the harm done to species 2. Observing unusual interactions with the opposite pattern, while low in number, should at least be briefly discussed, especially if they are used to support the central claim that interaction types are not detectable via. correlation methods. As a side note, perhaps the +/- class of interactions could be more adequately labeled “exploitative”, as they also include non-parasitic interactions such as predation.

(5) Response to reviewer

*The reviewer is right that our example of parasitism in the rebuttal is in fact a case of asymmetric competition; point well taken. We also agree with the reviewer that the +/– class of interactions are better labelled ‘exploitative’ rather than ‘parasitic’ interactions and we have changed this labelling throughout. However, the original point was that parasitic interactions are more stable when the exploitative benefit outweighs the negative effect, and that negative correlations are therefore unlikely because these were only observed in our simulations under opposite conditions. We partially concur with this interpretation: we observed negative correlations when both effects were of similar magnitude, but the variability in exploitative benefit was outweighed by the variability in the negative effect. To make this clearer, we have added the following to the Results section:*

*[line 377] “Correlations under* ***exploitative*** *interactions among bacteria, benefitting one but harming the other species, generally yielded positive correlations (Figs 2E, 2F and S6 Fig), but negative correlations* ***were*** *also found****. This happened*** *when* ***the exploitative benefit was of equal magnitude as the harm done to the other species (S6D Fig), or of similar mean magnitude but with more variation (e.g. species 1 is exploited by species 2; –α_12_ = α_21_*** ***and*** *σ_α12_ << σ_α21_ (****exploitative interaction type*** *1) or* ***species 2 is exploited by species 1; α_12_ = –α_21_*** ***and*** *σ_α21_ << σ_α12_ (****exploitative interaction type*** *2) (****S6B Fig****).* ***However, if the exploitative benefit outweighs the harm done to the other species, exploitative interactions will generally yield positive correlations.*** *It should* ***also*** *be noted that the two species were not exchangeable…”*

*In addition, we have added the following section to the Discussion:*

*[line 547] “Symmetric interaction types, where species involved affect each other’s growth in a qualitatively similar way (competition, mutualism) can be recovered, although competitive interactions may also result in positive correlations, albeit in very rare cases* ***where species have identical competitive strength. Likewise, we found that exploitative interactions generally induce positive correlations, especially in the likely circumstance where the exploitative benefit outweighs the harm to the exploited species. These findings*** *might explain why empirical correlation-based networks have a relative shortage of negative correlations (****Freilich et al. 2011;*** *Cougoul et al., 2019; Gupta et al., 2020). It* ***remains to be investigated*** *whether the high frequency of positive edges in reconstructed networks is caused by methodologic limitations or whether the interspecific interactions* ***in host-associated microbiota*** *are primarily mutualistic (Ma, 2018; Seelbinder et al., 2020;* *Röttjers and Faust, 2018****;*** ***Machado et al. 2021****].*

(6) Reviewer’s comment

with regard to author response 5: [regarding the adjusted abstract]: “limited information about the underlying web of interactions” sounds too strong, as many important characteristics of interaction networks (interaction signs, hub detection, various graph metrics) are either well recovered by correlations or have not been investigated by the authors. Suggestion: "limited information on specific interaction types” or “cannot distinguish detailed interaction types”. The line “competitive interactions may result in positive as well as negative correlations” is slightly misleading as is: requires quantification, as only very small fractions for competitive, commensal and amensal interactions had minority signs. Especially since this is already stated in the text (L. 530), it should for transparency also be in the abstract.

(6) Response to reviewer

*Following suggestions from the reviewer, we have rewritten the Abstract as follows:*

*[line 33] “The sign of a correlation mostly coincided with the nature of the strongest pairwise interaction, but this is not necessarily the case. For instance,* ***under rare conditions of identical interaction strength,*** *we found that competitive* ***and exploitative*** *interactions* ***can*** *result in positive as well as negative correlations. Thus, cross-sectional abundance data carry limited information* ***on specific interaction types****. Correlations in abundance may hint at interactions but require independent validation.”*

(7) Reviewer’s comment

with regard to author response 7: While the addition to the methods section helps clarity, it is easy to overlook. It would therefore be useful also have a condensed reference to the parameterization challenges in the caveat section of the Discussion section.

(7) Response to reviewer

*As suggested by the reviewer, we now reiterate the challenges of adequate model parametrization in the Discussion section, together with other limitations, as follows:*

*[line 650] “As a general critique, the use of simulated data based on gLV dynamics raises the question to what extent the necessary model assumptions (and therefore the results) are representative for the human microbiome. Of course, real data are much more complex than simulated data.* ***To reiterate, our base-case parametrization does not reflect any particular real-world system, and findings should be appreciated from a qualitative rather than quantitative viewpoint. Even so,*** *while models can only serve as very crude approximations, the main features of model-based analysis might still hold, as demonstrated by Freilich et al. (2018) [42].”*

**Addition by the authors:**

We noticed that the legend did not correspond to the correct panels of Fig. 2E and F and Fig. S6 (exploitative interaction type 1 and exploitative interaction type 2), therefore we corrected that. We also slightly changed the parameters of the distributions used to draw the plots in panels A, B and C of Fig. S6. This new set of parameters did not affect the main conclusions, but they illustrate the interaction types more clearly.
