# Supplementary material for: Species abundance correlations carry limited information about microbial network interactions
Source: PLoS Comput Biol. 2022 Sep 9;18(9):e1010491. doi: 10.1371/journal.pcbi.1010491 (PMC9518925; doi:10.1371/journal.pcbi.1010491)
Supplement: Attachment — Submitted filename: Rebuttal_PCOMPBIOL_12082022_2.docx [file pcbi.1010491.s012.docx]

Dr. Kiran Raosaheb Patil

Deputy Editor

*PLOS Computational Biology*

Leiden University Medical Center

Department of Biomedical Data Sciences

Einthovenweg 20

2333 ZC Leiden, The Netherlands

+31 6344 45 891

s.pinto@lumc.nl

Leiden, 12 August 2022

PCOMPBIOL-D-22-00053R3

Dear editors, dear Dr. Patil,

Thank you for giving us the opportunity to publish our manuscript entitled “Species abundance correlations carry limited information about microbial network interactions” in PLOS Computational Biology. Below is our response to the comment raised by the reviewer. In addition, we have corrected some slight typos that were still present in the latest version. These are visible in the new manuscript with tracked changes.

On behalf of all co-authors, I thank you for your help to revise and improve our manuscript.

Also on behalf of the co-authors,

Yours sincerely,

Susanne Pinto, MSc

***********************

**Reviewer's Responses**

Comments to the Authors:

(1) Reviewer’s comment

"...interactions are relatively rare among free-living bacteria and, if present, are often unidirectional. In contrast, Machado et al. (2021) suggested that" --> In contrast is not fully incorrect since free-living and host-associated communities have been shown to have different patterns. I think removing "in contrast" should fix it.

(1) Response to reviewer

*We thank the reviewer for the critical assessment and constructive feedback. We have changed the sentence as suggested by removing ‘In contrast, …’.*

*[line 625] “Based on metabolic modelling, Freilich et al. (2011) concluded that cooperative interactions are relatively rare among free-living bacteria and, if present, are often unidirectional. ~~In contrast,~~ Machado et al. (2021) suggested that mutualistic interactions are much more common among host-associated bacteria, that often form highly cooperative communities and have smaller genomes and fewer metabolic genes compared to other species.”*
